# Cream Formulated with Lemon Essential Oil-Loaded Pectin Capsules: Effects on Microbiological Quality and Sensory Properties

**DOI:** 10.3390/foods14162828

**Published:** 2025-08-15

**Authors:** Rofia Djerri, Salah Merniz, Louiza Himed, Maria D’Elia, Luca Rastrelli

**Affiliations:** 1 The Biotechnology and Food Quality Research Laboratory (BIOQUAL), Department of Food Biotechnology, Institute of Nutrition, Food and Agro-Food Technologies (INATAA), University Freres Mentouri Constantine 1, Route de Ain El-Bey, Constantine 25000, Algeria; rofia.djerri@student.umc.edu.dz; 2 Institute of Industrial Hygiene and Safety, University Batna 2, Batna 05078, Algeria; s.merniz@univ-batna2.dz; 3 Department of Pharmacy, University of Salerno, Via Giovanni Paolo II, 132, 84084 Salerno, Italy; mdelia@unisa.it; 4 National Biodiversity Future Center (NBFC), 90133 Palermo, Italy; 5 Dipartimento di Scienze della Terra e del Mare, University of Palermo, 90133 Palermo, Italy

**Keywords:** cream, pectin microcapsules, lemon essential oil, sensory analysis, microbiological stability

## Abstract

This study aimed to develop a novel cream formulation incorporating pectin-based microcapsules loaded with lemon essential oil (LEO), with the goal of enhancing both sensory attributes and microbiological quality. The capsules were added at increasing concentrations (0%, 0.25%, 0.5%, 0.75%, and 1%) to assess their impact. Physicochemical analysis revealed that higher capsule content significantly improved consistency and viscosity. Microbiological evaluations confirmed the absence of key foodborne pathogens, including *Salmonella* spp., *Listeria monocytogenes, Staphylococcus aureus*, and Enterobacteriaceae, in all formulations. Additionally, the antibacterial efficacy of the encapsulated LEO was validated against *Escherichia coli* and *Staphylococcus aureus* strains. Sensory analysis using paired comparison, ranking, and hedonic tests demonstrated a clear preference for samples enriched with the 0.5% and 0.75% capsules, noted for their enhanced creaminess, pleasant lemon aroma, and well-balanced flavour. Statistical analysis (ANOVA and principal component analysis, PCA) confirmed significant differences among samples, particularly in texture and aroma attributes. These findings highlight the potential of LEO-loaded pectin capsules as a clean-label strategy to improve both the sensory appeal and microbial safety of cream formulations.

## 1. Introduction

Dairy creams, both fermented and unfermented, are appreciated for their rich texture, nutritional value, and sensory appeal. Cream, commonly known as heavy or double cream, is a high-fat dairy product widely used in culinary applications due to its creamy consistency and its ability to enhance flavor and mouthfeel. However, modern consumers increasingly seek dairy products that go beyond basic nutrition, favoring those enriched with natural ingredients that provide enhanced sensory properties, shelf life, and microbiological safety [1,2,3].

A promising approach to address these expectations involves the incorporation of natural bioactive compounds, especially essential oils, which are well known for their antimicrobial and antioxidant potential [4,5]. Lemon essential oil (LEO), rich in limonene and other bioactive volatiles, is highly valued for its fresh citrus aroma and recognized biological activities [6,7]. Nevertheless, the direct addition of essential oils to dairy matrices presents formulation challenges due to their high volatility, low water solubility, and susceptibility to oxidation, which may compromise both product stability and sensory acceptability [8,9]. To overcome these limitations, encapsulation technologies have been developed to enhance the stability and controlled release of essential oils in food systems [10,11].

Pectin, a natural polysaccharide primarily derived from citrus peel, has emerged as an ideal encapsulating agent for hydrophobic compounds. Its biocompatibility, gelling properties, and ability to form stable matrices make it well suited for the microencapsulation of essential oils [11,12]. Pectin-based encapsulation not only protects essential oils from environmental degradation but also facilitates their gradual release, minimizing sensory overload and preserving functionality during food processing and storage [13,14]. In our recent study, ultrasound-enhanced ionotropic gelation has improved the encapsulation efficiency and structural integrity of pectin-based capsules containing LEO, with promising results in fresh-cut fruit preservation [11]. In that work, lemon essential oil encapsulated in pectin matrices significantly reduced browning and microbial load in apple slices during storage, demonstrating the dual function of aroma retention and active food protection.

Despite these advances, the integration of encapsulated EOs into dairy products, particularly cream-based matrices, remains underexplored. The high-fat content and complex rheology of creams pose specific formulation challenges but also offer opportunities for functional enhancement through microcapsule incorporation. Pectin-based capsules may help stabilize cream structure, reduce phase separation, and improve organoleptic properties, such as texture and aroma. Furthermore, the antimicrobial action of LEO may contribute to improved microbiological stability, supporting shelf life extension without the use of artificial preservatives.

This study aims to evaluate the incorporation of ultrasound-assisted pectin capsules loaded with lemon essential oil into fermented-free cream. Specifically, it investigates the impact on physicochemical parameters, microbial stability, and sensory perception through structured sensory analysis (paired comparison, ranking, and hedonic tests). Building upon our previous findings in fruit systems [11], this work seeks to extend the application of LEO encapsulation to dairy matrices, offering a clean-label solution for value-added cream products in line with consumer demand and sustainability trends.

## 2. Materials and Methods

### 2.1. Application of Encapsulated Essential Oils to Set-Style Fresh Cream

This study investigated the impact of pectin-based microcapsules loaded with lemon essential oil (LEO) on the quality attributes of set-style cream prepared without lactic acid bacterial fermentation. The aim was to explore a clean-label strategy to enhance both microbiological stability and sensory performance through the integration of functional encapsulated bioactives. The entire formulation process was conducted under rigorously controlled hygienic conditions to prevent microbial contamination and to preserve the integrity of the encapsulated system. The process was carried out in a pilot-scale dairy facility, where temperature, humidity, and equipment sterilization were monitored and documented throughout production. The experimental design focused on ensuring minimal disruption of capsule morphology during incorporation into the cream matrix. To this end, LEO-loaded pectin capsules were gently dispersed into the cream post-pasteurization and prior to cold-setting, at concentrations of 0%, 0.25%, 0.5%, 0.75%, and 1% (*w*/*w*), based on preliminary optimization studies. The capsules were incorporated into the cream once its temperature reached 20–25 °C, during the post-pasteurization cooling phase, and stirred at low speed (50 rpm) using a sterile overhead paddle stirrer to avoid rupture or premature diffusion of essential oil. The cream base itself was formulated using high-fat pasteurized milk cream (minimum 35% fat), without the addition of lactic acid bacteria or emulsifiers, to highlight the independent effects of the encapsulated essential oil system. After capsule incorporation, the cream was immediately packaged in sterile, light-impermeable polypropylene containers and stored at 4 ± 1 °C until further analysis. This protocol was developed to facilitate the controlled release of LEO from the capsule matrix over storage time, allowing evaluation of its contribution to microbial suppression and sensory modification. The structural preservation of capsules within the cream matrix was confirmed via preliminary optical microscopy analysis, and no significant capsule rupture was observed under cold storage. The subsequent analytical steps included physicochemical measurements (viscosity, syneresis, and pH), microbiological assays (pathogen screening and total viable counts), and sensory evaluation (hedonic scale, preference ranking, and PCA-based mapping), as detailed in the following sections.

### 2.2. Raw Milk Reception and Preliminary Quality Assessment

Upon arrival at the pilot dairy facility, raw bovine milk underwent standardized quality control procedures to ensure suitability for processing. Initial screening included pH determination using a calibrated pH meter (Hanna Instruments, (Ronchi Di Villafranca Padovana, Italy)) and antibiotic residue detection employing a rapid screening test (e.g., Delvotest^®^ T or equivalent), in compliance with EU Regulation (EC) No. 853/2004 for raw milk intended for human consumption. Only batches that met the acceptance criteria, i.e., pH values between 6.6 and 6.8 and absence of antibiotic residues, were admitted for further processing. Rejected batches were documented and excluded from the study. Approved milk was weighed using a calibrated industrial scale and transferred to sanitized stainless-steel holding tanks, where it was maintained at 4 ± 1 °C to prevent microbial growth and enzymatic degradation prior to cream separation. Continuous agitation ensured homogeneity and minimized cream line formation during short-term storage (<12 h). All handling procedures adhered to HACCP-based protocols for milk hygiene and traceability. This initial stage was critical for ensuring the compositional and microbiological integrity of the final cream matrix, particularly given the absence of starter cultures and the reliance on essential oil encapsulates for microbial stability.

### 2.3. Preparation of Lemon Essential Oil-Loaded Pectin Capsules

Encapsulated systems were developed using the ionotropic gelation technique to entrap lemon essential oil (LEO) within a calcium-crosslinked pectin matrix. Low methoxyl pectin (degree of esterification < 50%) was selected for its strong gelling behavior in the presence of divalent cations, particularly Ca^2+^.

A 2% (*w*/*v*) aqueous solution of low methoxyl pectin was prepared by gradual dispersion under magnetic stirring (600 rpm, 30 min) at ambient temperature. After complete solubilization, lemon essential oil was added at four final concentrations, namely 0.25%, 0.5%, 0.75%, and 1% (*w*/*w*) relative to the total volume. The emulsion was homogenized using a high-shear Ultra-Turrax^®^ T25 digital homogenizer (IKA, Staufen, Germany) at 12,000 rpm for 5 min to ensure fine droplet dispersion and stabilization within the biopolymer matrix.

The resulting oil-in-water emulsion was then extruded dropwise into a gently stirred 100 mM calcium chloride (CaCl_2_) solution using a syringe pump (needle diameter: 0.5 mm). Ionotropic gelation occurred upon contact, resulting in the rapid formation of spherical gel beads. Capsules were allowed to harden in the calcium bath for 30 min, then filtered and gently rinsed with deionized water to remove surface-bound ions.

The capsules were stored in sterile containers at 4 ± 1 °C in hydrated form and used within 48 h of preparation. A corresponding batch of empty control capsules (without LEO) was produced under identical conditions and served as a negative control in both microbiological and sensory analyses.

**Figure 1:**
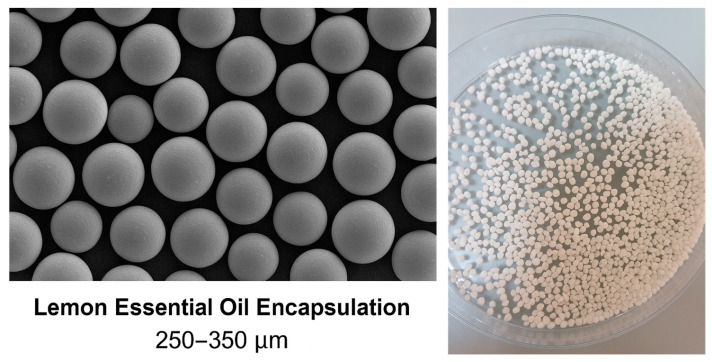
Optical microscopy image of lemon essential oil-loaded pectin capsules obtained by ultrasound-assisted ionotropic gelation. Capsules appear spherical and uniform, with an average diameter ranging from 250 to 350 µm. The encapsulation process preserved morphological integrity and is suitable for incorporation into dairy matrices.

The structural integrity and morphology of the capsules were confirmed via light microscopy, and their average diameter was approximately 250–350 µm. This method was selected for its mild processing conditions, food-grade safety, and ability to encapsulate volatile and thermolabile compounds without chemical crosslinkers or organic solvents (Figure 1).

### 2.4. Preparation of Set-Style Fresh Cream

**Figure 2:**
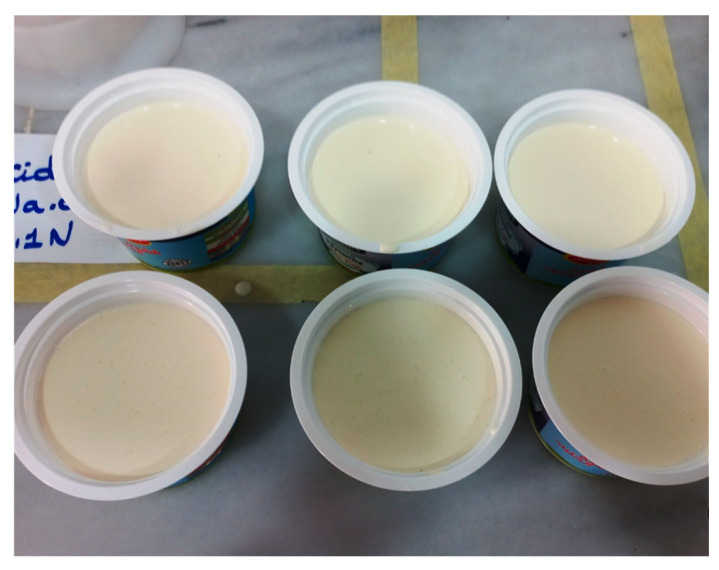
Visual appearance of cream samples before the incorporation of lemon essential oil-loaded pectin capsules. The creams exhibit a smooth, uniform surface and consistent opacity, reflecting good emulsion stability and absence of phase separation prior to enrichment.

The manufacturing process of set-style fresh cream was conducted through a standardized protocol aimed at ensuring both microbial safety and structural suitability for the incorporation of lemon essential oil (LEO)-loaded pectin capsules. Each stage of the process was optimized to preserve the functional integrity of the microcapsules while enabling a stable and organoleptically acceptable cream matrix (Figure 2).

Step 1: Raw Milk Pasteurization

Raw cow’s milk was pasteurized at 80 °C for 15 s using a continuous-flow plate heat exchanger. This high-temperature short-time (HTST) treatment was chosen to eliminate pathogenic and spoilage microorganisms while preserving the milk’s protein structure and nutritional profile, as supported by Jayarao et al. [15].

Step 2: Cream Separation

Following pasteurization, centrifugal skimming was employed to separate cream from skim milk using a mechanical cream separator. The resulting cream fraction contained approximately 30–40% milk fat, consistent with industry standards for cream production.

Step 3: Fat Standardization

The separated cream was then standardized to 30% milk fat, adjusted using skimmed milk to meet the desired specifications for set-style cream. This step ensures consistency across batches and influences textural and rheological properties.

Step 4: Secondary Pasteurization of Cream

The standardized cream underwent a second pasteurization step at 90 °C for 20 s to ensure microbial stability in the absence of lactic acid bacteria or other bioprotective cultures, following the conditions reported by Machado et al. [16]. This treatment also contributed to the partial denaturation of whey proteins, which enhanced cream viscosity and emulsion stability.

Step 5: Cooling

Immediately after heat treatment, the cream was rapidly cooled to 20–25 °C to minimize microbial growth and enzymatic degradation. This temperature range was selected as optimal for the subsequent incorporation of pectin capsules, preserving their structural and functional integrity.

Step 6: Incorporation of Pectin Capsules

Pectin-based capsules containing lemon essential oil were added to the cooled cream at five graded concentrations, namely 0% (control), 0.25%, 0.5%, 0.75%, and 1% *w*/*w*. Incorporation was performed under gentle mechanical agitation (50 rpm) using a sterile paddle mixer to ensure homogenous dispersion while avoiding capsule rupture or premature oil release. The incorporation occurred after the pasteurization step, ensuring that any bioactive effects could be specifically attributed to the encapsulated oil.

Step 7: Packaging

The enriched cream was filled into pre-sterilized polypropylene containers (120 mL), sealed under hygienic conditions using a semi-automatic lidding machine. A tamper-evident seal was applied to ensure product integrity and minimize post-process contamination. Products were labelled with a recommended shelf life and refrigeration instruction.

Step 8: Cold Storage and Maturation

Packaged cream samples were stored at 4 ± 1 °C throughout the analysis period. Time-point evaluations were conducted on days 0, 3, 7, 10, 14, 21, and 28 to assess microbiological load, physicochemical stability (pH, viscosity, and syneresis), and sensory quality.

### 2.5. Antibacterial Activity Assay

#### 2.5.1. Microorganisms and Culture Conditions

Two reference bacterial strains were employed to evaluate the antibacterial efficacy of lemon essential oil-loaded pectin capsules, namely *Escherichia coli* ATCC 25922 (Gram-negative) and *Staphylococcus aureus* ATCC 25923 (Gram-positive). Each strain was subcultured in Mueller–Hinton broth (MHB) and incubated at 37 ± 1 °C for 18–24 h under aerobic conditions. Bacterial suspensions were standardized to a 0.5 McFarland turbidity standard, corresponding to approximately 1 × 10^8^ CFU/mL, using a nephelometer Densimat^®^ (bioMérieux, Marcy l’Etoile, France).

#### 2.5.2. Agar Well Diffusion Method

The antibacterial activity of the encapsulated formulations was assessed using the agar well diffusion assay, following CLSI guidelines (M100). Mueller–Hinton agar (MHA) plates were uniformly inoculated with the standardized bacterial suspensions using sterile cotton swabs to ensure even coverage.

Wells of 6 mm diameter were aseptically punched into the agar using a sterile cork-borer. Each well was filled with 100 µL of capsule suspension, corresponding to approximately 10 hydrated capsules dispersed in sterile phosphate-buffered saline (PBS). The following treatments were evaluated: test (capsules containing LEO at 0.25%, 0.5%, 0.75%, or 1% (*w*/*w*)), positive control (neat lemon essential oil (100 µL)); negative control (capsules prepared without essential oil (blank)).

Plates were incubated at 37 ± 1 °C for 24 h, after which the zones of inhibition were measured using a digital caliper with ±0.1 mm precision. All experiments were conducted in triplicate, and results are expressed as mean inhibition diameter ± standard deviation (mm).

This method enabled a comparative evaluation of the antimicrobial potential of free and encapsulated lemon essential oil against clinically relevant bacterial targets and provided insight into the protective and controlled-release effects of the pectin capsule matrix.

### 2.6. Stability Monitoring of the Formulated Cream

Comprehensive physicochemical characterization of the cream samples was performed to assess the influence of LEO-loaded pectin capsules on product stability, composition, and quality. All analyses were conducted in triplicate, and mean values ± standard deviation were reported. The physicochemical parameters, including pH, titratable acidity, fat content, total solids, and density, were measured following the procedures and quality standards described by Burke et al. [17], which outline analytical protocols commonly applied in dairy process monitoring.

#### 2.6.1. pH Measurement

The pH of each cream sample was determined at 20–25 °C using a calibrated digital pH meter equipped with a combined pH/temperature probe (Hanna Instruments^®^, model HI 5221). The probe was immersed directly into homogenized samples, and readings were recorded after stabilization [17].

#### 2.6.2. Fat Content Determination

Fat content was measured using the Gerber acid butyrometer method. In this technique, sulfuric acid is added to digest proteins and release fat, followed by isoamyl alcohol to facilitate fat separation. The sample was centrifuged at 65 °C for 10 min, and fat content was read on a calibrated butyrometer [17].

#### 2.6.3. Titratable Acidity

Titratable acidity (TA) was determined by alkaline titration using N/9 sodium hydroxide (NaOH) and phenolphthalein as an indicator. The volume of NaOH required to reach the endpoint (pink color persistence) was recorded. Acidity was expressed in Dornic degrees (°D), where 1°D = 0.1 mL of N/9 NaOH per 100 mL of sample [18].

#### 2.6.4. Total Solids Content

The total solids content (dry matter) was determined gravimetrically by drying approximately 5 g of homogenized cream in an oven at 125 °C for 15 min, followed by equilibration in a desiccator until constant weight [17]. Moisture content was calculated by difference.

#### 2.6.5. Density Measurement

Cream density was measured at 20 °C using a calibrated thermo-lacto-densimeter, with correction factors applied for temperature deviations. This method followed the protocol described by Borba et al. [19] and was used to evaluate sample homogeneity and composition shifts due to capsule inclusion.

#### 2.6.6. Determination of Apparent Viscosity

Apparent viscosity was assessed at 4 °C using a rotational viscometer Brookfield DV-E, spindle No. 4, 50 rpm (Middleboro, MA USA), in accordance with Gün et al. [20]. Each measurement was conducted after allowing the sample to equilibrate to the test temperature, and results were expressed in mPa·s.

### 2.7. Microbiological Analyses

Microbiological quality assessments of the cream samples were performed to verify compliance with national food safety criteria and to assess the efficacy of lemon essential oil-loaded pectin capsules in controlling pathogenic microorganisms. All analyses were conducted in accordance with the Interministerial Decree of October 4, 2016 regarding microbiological criteria for foodstuffs (JORA No. 64, 2016), and consistent with ISO methodologies where applicable. Enumeration of *Enterobacteriaceae* was carried out according to ISO 21528-1:2017 and ISO 21528-2:2017 (Colony Count Technique) [21,22]. Results were expressed as CFU/g. Quantification of *Staphylococcus aureus* and related coagulase-positive strains was performed using Baird–Parker agar supplemented with egg yolk tellurite, [23,24,25]. Presumptive colonies were confirmed by coagulase testing. The presence of *Salmonella* spp. was investigated in 25 g of sample. The method included (i) pre-enrichment in buffered peptone water; (ii) selective enrichment in Rappaport–Vassiliadis and Muller–Kauffmann tetrathionate broth; (iii) plating on selective agars (e.g., XLD, Hektoen); (iv) biochemical and serological confirmation [26]. Detection of *Listeria monocytogenes* was performed using a two-step enrichment method (Half-Fraser and Fraser broths), followed by isolation on selective media (e.g., PALCAM or ALOA) and confirmation via biochemical assays [27]. All analyses were conducted at each storage time point (days 0, 3, 7, 10, 14, 21, and 28), and results were compared across treatments to assess the inhibitory effects of LEO encapsulation.

### 2.8. Sensory Analysis

#### 2.8.1. Panel Composition

The sensory analysis of cream formulations enriched with lemon essential oil-loaded pectin capsules was conducted in a sensory laboratory A trained panel of 10 dairy experts (5 males and 5 females, aged between 25 and 55 years), from Safilait’s Dairy R&D Unit participated in the evaluation. All participants provided written informed consent prior to the evaluation, and the procedure was approved by the Director of the Bio-technology and Food Quality Research Laboratory (BIOQUAL), INATAA, University of the Brothers Mentouri Constantine 1, Algeria (March 2024). All panelists were selected and trained according to EN ISO 8586:2014-03, the international standard for the selection and training of sensory assessors [28]. Sensory assessments were performed under standardized conditions (lighting, temperature, and ventilation) to minimize external influences and ensure consistency. The profiling method followed the guidelines of ISO 13299:2016-05E [29], using a 5-point descriptive intensity scale, where 1 indicated no perception of the attribute and 5 indicated very high intensity. Panelists were equipped with definition cards and score sheets covering key sensory descriptors relevant to dairy matrices, including texture, aroma, flavor, mouthfeel, and overall impression.

#### 2.8.2. Paired Comparison Test

The paired comparison test was employed to determine whether statistically significant differences existed between sample pairs regarding the sensory attribute of aroma, particularly the presence and intensity of lemon notes introduced by LEO capsules [30].

Ten combinations of paired samples were prepared from the five treatments (0%, 0.25%, 0.5%, 0.75%, and 1% capsule inclusion), coded as follows: AB, AC, AD, AE, BC, BD, BE, CD, CE, DE, respectively. Each pair was presented in randomized order using coded, identical cups (20 mL/sample). Panelists were instructed to rinse their mouths with room-temperature water between samples.

Each panelist recorded whether they perceived a difference in aroma and indicated the preferred sample, if applicable. Results were analyzed using the chi-square (χ^2^) test, comparing observed frequencies with theoretical expectations to assess statistical significance at *p* < 0.05.

#### 2.8.3. Ranking Test Procedure

To evaluate overall consumer acceptability, a ranking test was conducted in which the five cream samples (0%, 0.25%, 0.5%, 0.75%, and 1%) were presented simultaneously in a randomized order. Panelists were instructed to rank the samples from least to most acceptable based on overall impression, considering flavor, texture, and aroma.

Each sample (coded and anonymized) was presented in an individual cup, and panelists were allowed to retaste freely before final ranking. The total scores for each sample were calculated by summing the ranks assigned by all panelists (lowest score = most preferred).

Statistical differences between samples were evaluated using Friedman’s test, a non-parametric method suitable for ordinal sensory data. Post hoc pairwise comparisons were performed when required using the Nemenyi test, with significance set at *p* < 0.05.

#### 2.8.4. Hedonic Test

A 9-point hedonic scale test was employed to evaluate the overall sensory acceptability of the sour cream samples from the perspective of trained assessors. This method allowed for the generation of a multidimensional sensory profile by capturing panellists’ affective responses to specific product attributes.

Each panelist received all five coded cream samples simultaneously (A: 0%, B: 0.25%, C: 0.5%, D: 0.75%, and E: 1%) presented in identical, anonymized containers (20 mL per sample). Samples were randomized and equilibrated to 8–10 °C before evaluation. Panelists were instructed to rate each sample on a 9-point hedonic scale ranging from 1 (“dislike extremely”) to 9 (“like extremely”) across five sensory parameters, namely texture, color, odor, taste, and aroma.

All panelists completed standardized evaluation forms under controlled testing conditions with palate cleansing between samples.

The sensory scores were subjected to analysis of variance (ANOVA) using XLSTAT software (version 2010) to determine statistically significant differences (*p* < 0.05) among samples. When significance was detected, principal component analysis (PCA) was applied to the dataset to explore correlations between sample composition and sensory descriptors. PCA enabled the visualization of attribute clustering and the differentiation of treatments based on their perceptual profiles.

The results provided insights into how varying concentrations of lemon essential oil-loaded pectin capsules influenced consumer-relevant attributes, such as creaminess, lemon aroma, and overall liking.

### 2.9. Statistical Analysis

All experimental data were expressed as the mean ± standard deviation (SD) of at least three independent replicates. Statistical analysis was performed using XLSTAT software (version 2010) and GraphPad Prism (version 8.0) for graphical visualization and hypothesis testing.

For physicochemical and microbiological parameters, differences between treatments were assessed using one-way analysis of variance (ANOVA), followed by Tukey’s HSD post hoc test to determine pairwise significance at a 95% confidence level (*p* < 0.05).

For sensory evaluation:

• Paired comparison data were analyzed using the chi-square (χ^2^) test.
• Ranking test results were analyzed using Friedman’s test, a non-parametric test for related samples.
• Hedonic test scores were processed by ANOVA, and significant effects were further examined using PCA.

Principal component analysis (PCA) was employed to identify correlations between sensory descriptors and sample treatments. Multivariate analysis enabled data dimensionality reduction and visualization of perceptual differences among formulations. Sensory and analytical data were standardized prior to PCA using the correlation matrix method.

A significance threshold of *p* < 0.05 was adopted for all tests unless otherwise specified.

## 3. Results

### 3.1. Incorporation of Encapsulated Essential Oils into Cream

The incorporation of lemon essential oil-loaded pectin capsules was successfully carried out during the post-pasteurization cooling phase, once the cream temperature fell to approximately 20–25 °C, a range that preserved the structural integrity of the capsules while allowing for uniform distribution in the cream matrix.

This approach was adopted to preserve both the structural integrity of the capsules and the stability of thermolabile volatile compounds, such as limonene and citral, which are highly susceptible to degradation through heat and oxidation [31,32]. Preliminary experiments demonstrated that incorporating the capsules prior to pasteurization significantly diminished both the intensity of the lemon aroma and the antimicrobial efficacy of the final product. These effects were attributed to the volatilization and oxidative breakdown of essential oil constituents at high temperatures, as well as to the partial depolymerization of the pectin matrix, which typically occurs above 70 °C, thereby compromising the integrity of the encapsulating network and altering its release profile [33].

Conversely, the addition of capsules after thermal treatment effectively maintained their morphological integrity and facilitated a more controlled release of the active compounds during storage. Sensory and microbiological data, presented in the subsequent sections, further confirmed that post-pasteurization incorporation not only preserved the functional attributes of the essential oil but also contributed to improved homogeneity of dispersion, enhanced texture and creaminess, and a more pronounced perception of freshness and lemon aroma. These observations are consistent with previous findings that highlight the advantages of post-processing inclusion of essential oil delivery systems to avoid thermal degradation and maintain bioactive performance [34,35].

### 3.2. Antibacterial Activity of Lemon Essential Oil-Loaded Capsules

The antibacterial properties of lemon essential oil (LEO)-loaded pectin capsules were evaluated against two reference strains: *Escherichia coli* (ATCC 25922) and *Staphylococcus aureus* (ATCC 25923). The results demonstrated a clear dose-dependent inhibitory effect for both Gram-negative and Gram-positive bacteria. No inhibition zones were detected in the control group (0% LEO), confirming that the pectin capsules alone lacked antibacterial activity.

As LEO concentration increased from 0.25% to 1.0% within the capsules, the diameter of the inhibition zones progressively expanded, indicating enhanced antimicrobial efficacy. The highest antibacterial activity was observed at 1.0% LEO, with inhibition zones reaching 15.1 ± 0.4 mm for *E. coli* and 18.9 ± 0.5 mm for *S. aureus*. These values approached those obtained with pure LEO, which served as the positive control, further confirming the preserved activity of the encapsulated essential oil.

Notably, *S. aureus* appeared more sensitive to the LEO formulations than *E. coli*, consistent with the previous literature reporting the higher susceptibility of Gram-positive bacteria to essential oils due to differences in cell wall structure. The data underscore the potential of encapsulated LEO to serve as a natural antimicrobial agent suitable for application in dairy matrices. Complete results are reported in Table 1.

### 3.3. Physicochemical Properties of the Formulated Cream

#### 3.3.1. Milk Density

The raw milk used in this study exhibited a density of 1029 ± 0.96 kg/m^3^, which falls within the standard range reported for bovine milk (1028–1032 kg/m^3^). Density is influenced by the content of dry matter, fat content, temperature, and animal feeding practices [36]. In particular, a higher concentration of non-fat solids is associated with increased density values [37], supporting the quality and suitability of the raw material for cream production.

#### 3.3.2. Total Solids (Dry Matter)

The total solids content of raw milk was 12.5 ± 0.75%. During cream processing, a slight decrease in total solids was observed after pasteurization (from 37.85 ± 0.12% to 37.60 ± 0.10%), which may be attributed to thermal rearrangement of protein structures and minor water redistribution or loss during heat treatment [38,39]. Following capsule incorporation, total solids increased progressively with rising capsule concentrations, reaching 39.10 ± 0.14% at 1.0% inclusion (Table 2). This trend reflects the added dry matter contributed by both pectin and encapsulated lemon essential oil, which structurally enrich the cream matrix. The observed increment aligns with previous studies in encapsulated systems, where biopolymer matrices enhance product consistency, richness, and mouthfeel through water-binding and volume-filling effects [40].

#### 3.3.3. Fat Content

The raw milk had a fat content of 4 ± 0.3%, which, after centrifugal skimming, yielded cream with a fat content of 30 ± 5%. Notably, the fat content remained stable throughout pasteurization and capsule incorporation, indicating that the encapsulation process did not interfere with lipid separation or stability.

#### 3.3.4. pH and Titratable Acidity

The initial pH and titratable acidity of raw milk were measured at 6.68 and 17 °D, respectively. Following pasteurization, the pH slightly decreased to 6.58, while acidity rose to 17.0 °D, indicating mild acidification, likely due to heat-induced protein unfolding and exposure of acidic amino acid residues [41]. Subsequent incorporation of LEO-loaded pectin capsules led to a gradual and consistent decrease in pH, reaching 6.35 at the highest capsule concentration (1%), accompanied by a parallel increase in titratable acidity to 19.0 °D (Table 2). These variations can be attributed to the intrinsic acidic properties of pectin and lemon essential oil compounds, such as citral and limonene derivatives. These acidification trends are beneficial for matrix structuring, as slight pH reductions near the isoelectric point of caseins can promote tighter protein networks and enhance water retention. Consequently, the observed pH–acidity profile may have contributed to improved cream viscosity, texture, and overall sensory perception.

#### 3.3.5. Apparent Viscosity

Apparent viscosity, measured at 4 °C, showed a progressive increase both after pasteurization and following the incorporation of lemon essential oil-loaded pectin capsules (Table 2). The initial rise in viscosity post-pasteurization (from 1230 ± 15 to 1315 ± 18 mPa·s) can be attributed to protein denaturation and partial unfolding, which promote intermolecular aggregation and the formation of a denser microstructure. Subsequent increases in viscosity were observed with capsule inclusion, notably at concentrations of 0.5% and above. At 1.0% capsule concentration, viscosity reached 1920 ± 28 mPa·s, representing a ~56% increase relative to the baseline. This trend reflects the gelling and water-binding capacity of low methoxyl pectin as well as the physical structuring effect of the capsules themselves, which likely act as particulate fillers that restrict flow and reinforce the matrix. These results are in agreement with previous studies on encapsulated systems in dairy formulations, where biopolymer-based particles contributed to enhanced texture and shear resistance due to increased phase cohesion and filler–matrix interaction [42]. (Table 2).

### 3.4. Stability Monitoring of the Formulated Cream

The storage stability of the formulated cream was monitored over a 28-day period under refrigeration and compared to a control sample without capsules. The results are summarized in Table 3.

The pH of the control cream decreased steadily from 6.58 at day 0 to 5.4 by day 28, suggesting microbial acidification, likely due to the activity of residual lactic acid bacteria or spoilage organisms. In contrast, the formulated cream containing lemon essential oil-loaded pectin capsules showed greater pH stability, with values ranging from 6.50 to 6.25, indicating enhanced preservation of acid–base balance.

Viscosity data revealed a notable decline in the control sample, decreasing from 1230 to 800 mPa·s over 28 days, accompanied by visible phase separation starting on day 7 and becoming severe by day 28. The formulated cream, however, retained better viscosity throughout storage, showing only a moderate decrease from 1450 to 1300 mPa·s. This indicates improved textural resilience, likely due to the water-binding and network-stabilizing effect of the pectin capsule matrix.

Phase separation behavior also differed substantially. While the control began to exhibit slight separation by day 7, progressing to marked aqueous phase separation by day 21 and severe destabilization by day 28, the formulated cream maintained homogeneity until day 21. Only by day 28 was slight to moderate separation observed, underscoring the structural stabilizing effect of the capsules.

From a microbiological perspective, *Enterobacteriaceae* counts remained consistently below 10 CFU/g in the formulated cream throughout the entire shelf life, while the control sample exceeded this threshold after day 14, reaching >10 CFU/g by day 21. A similar trend was observed for coagulase-positive *Staphylococci*, which increased to 150 CFU/g in the control by day 28, compared to just 50 CFU/g in the treated sample.

Although the pasteurization process effectively reduced the microbial load to below detection levels, the appearance of *Enterobacteriaceae* and *Staphylococcus aureus* during storage suggests the possibility of minor post-pasteurization contamination. This may have arisen from environmental exposure or manual handling during capsule incorporation and packaging. Future improvements to the process should consider implementation of a more controlled environment (e.g., laminar airflow or aseptic conditions) to mitigate this risk.

These differences support the antimicrobial efficacy of the lemon essential oil microcapsules. Importantly, both *Salmonella* spp. and *Listeria monocytogenes* were undetected in all samples and at all time points, indicating that baseline hygienic processing conditions were well maintained.

It is important to note that the lemon essential oil-loaded pectin capsules were incorporated after the pasteurization process. Therefore, the observed reduction in microbial growth may be associated with the presence of encapsulated essential oil, although the extent of its migration into the cream matrix was not quantified in this study. The formulated cream demonstrated significantly greater physical and microbiological stability during refrigerated storage compared to the control. The inclusion of LEO-loaded pectin capsules effectively preserved viscosity, delayed phase separation, limited microbial growth, and stabilized pH, thus enhancing the overall shelf life and safety profile of the product. While the lower microbial counts observed in the treated samples are consistent with a potential antimicrobial contribution of the lemon essential oil, this effect cannot be conclusively demonstrated in the absence of an intentional microbial challenge. Further studies, including challenge tests with specific bacterial strains and quantitative analysis of oil migration into the cream matrix (e.g., via GC-MS), are warranted to confirm this mechanism.

### 3.5. Sensory Evaluation

#### 3.5.1. Paired Comparison Test

To evaluate the perceptibility of sensory differences between cream formulations enriched with lemon essential oil-loaded pectin capsules, a paired comparison test was conducted using five coded samples, namely A (0%, control), B (0.25%), C (0.5%), D (0.75%), and E (1%). Ten trained panelists evaluated each pair in a randomized order under blinded conditions to minimize bias and focus the assessment on aroma-related distinctions.

Statistical analysis of the panelists’ responses using the chi-square (χ^2^) test revealed that all calculated χ^2^ values were below the critical value of 9 at the 5% significance level (α = 0.05), indicating the absence of statistically significant differences among all tested sample pairs. These findings suggest that, despite the progressive addition of encapsulated essential oil, the formulations remained sensorially homogeneous in terms of aroma detection. From a product development standpoint, this sensory uniformity is favorable, as it confirms that the functional encapsulation strategy does not detract from the baseline organoleptic quality, nor does it introduce disruptive aroma intensities (Table 4).

#### 3.5.2. Ranking Test Results

The ranking test was employed to assess the overall acceptability of the five cream samples (A–E) based on sensory descriptors, such as texture, taste, aroma, and general preference. Each panelist ranked the samples from 1 (most preferred) to 5 (least preferred). The results showed clear trends in sensory preference (Table 5).

**Figure 3:**
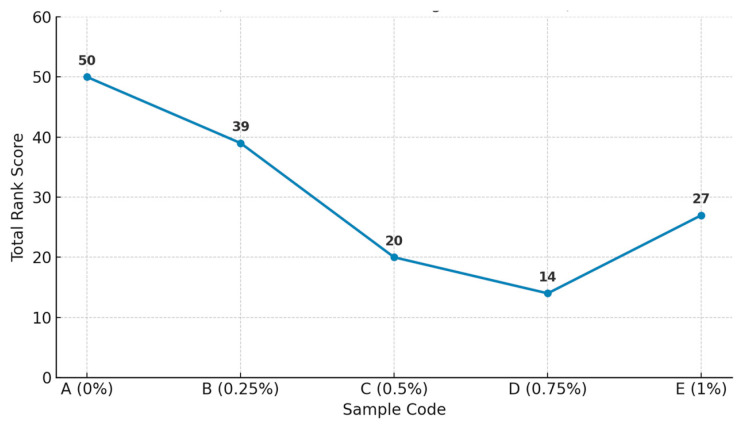
Ranking test results showing total scores for each cream formulation (lower scores indicate higher preference).

Sample A (control) received the highest cumulative rank score (50), indicating the lowest acceptability among panelists. Conversely, sample D (0.75% LEO capsules) was the most preferred, receiving the lowest score (14), followed by sample C (0.5%) with 20 and sample E (1%) with 27. Sample B (0.25%) ranked intermediately (39). The results highlight a progressive increase in acceptability with the addition of lemon-scented microcapsules, peaking at 0.75%, beyond which a slight decline was observed. This suggests that while encapsulation improved sensory appeal, an excessive concentration may lead to overpowering aroma or slight textural imbalance (Figure 3).

The ranking data were statistically significant (*p* < 0.05), confirming differences in panelist preferences. These findings validate the positive sensory impact of the capsules, particularly at intermediate concentrations, and support their use as a functional enhancement in non-fermented dairy products

#### 3.5.3. Sensory Evaluation

A 9-point hedonic test was conducted to assess the overall acceptability of cream samples enriched with increasing concentrations of lemon essential oil-loaded pectin capsules, i.e., A (0%, control), B (0.25%), C (0.5%), D (0.75%), and E (1%). Each sample was evaluated by trained panelists based on key sensory parameters, including texture (smoothness, creaminess, and fluidity), color (yellow hue), odor (fresh cream, lemon, herbal notes, and rancid), taste (sweet, acidic, bitter, and salty), and aroma (lemon, hazelnut, dried fruit, and raw milk). The evaluation was performed by a trained panel of 10 individuals. The complete hedonic test form used for the sensory evaluation is provided in the Supplementary Material (Tables S1–S3).

Statistical analysis using ANOVA (*p* < 0.05) revealed that specific attributes, such as smoothness, creaminess, and lemon aroma, varied significantly among the samples, indicating that capsule enrichment led to perceptible improvements in sensory quality. These attributes were most pronounced in samples C through E, suggesting a concentration-dependent effect of the encapsulated essential oil.

The sensory enhancement is attributed to two primary effects, namely (1) the gelling properties of pectin, which contribute to matrix cohesion and smoother mouthfeel; and (2) the controlled release of lemon essential oil, which provides a pleasant, progressively more intense aroma as capsule concentration increases. These combined effects led to greater panelist preference across multiple sensory dimensions.

**Figure 4:**
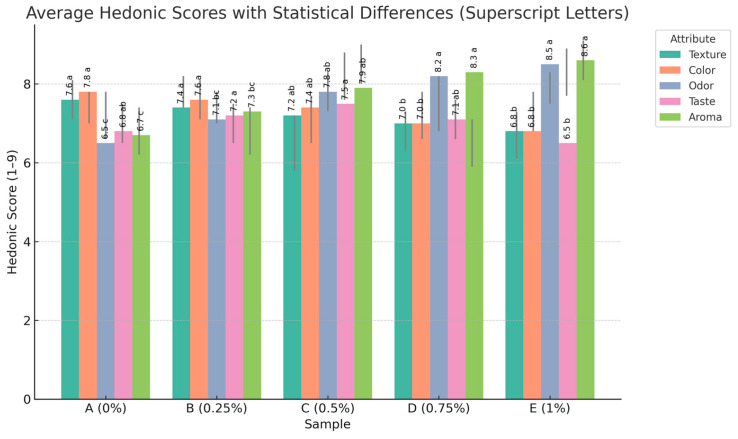
Hedonic scores (mean ± SD, *n* = 10) for cream samples enriched with increasing concentrations of pectin-based microcapsules containing lemon essential oil. Samples A (0%), B (0.25%), C (0.5%), D (0.75%), and E (1%) were evaluated by a trained sensory panel using a 9-point scale (1 = dislike extremely; 9 = like extremely). Different letters above the bars indicate statistically significant differences among means (*p* < 0.05). The complete hedonic test form is provided in the Supplementary Material.

**Figure 5:**
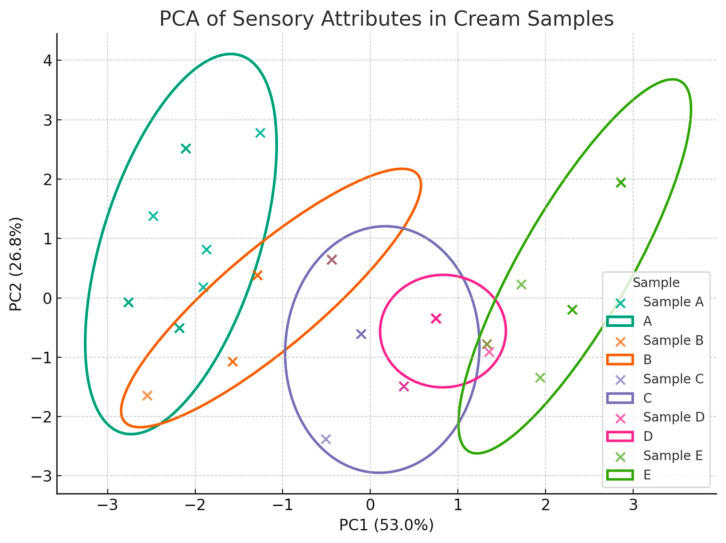
Principal component analysis (PCA) biplot of sensory attributes for cream samples enriched with increasing concentrations of lemon essential oil-loaded pectin capsules (A = 0%, B = 0.25%, C = 0.5%, D = 0.75%, and E = 1%). Each point represents the sensory evaluation of one trained panelist (*n* = 10). The 95% confidence ellipses indicate the clustering tendency for each formulation. The first two principal components (PC1 and PC2) explain 53.0% and 26.8% of the total variance, respectively. Samples C, D, and E clustered closely, associating with favorable sensory attributes, suggesting improved acceptability with higher capsule concentrations.

Panelists’ average scores on the 9-point hedonic scale further confirmed this trend (Figure 4). Sample A (control) scored 5.2, reflecting neutral to moderate appreciation. Sample B improved to 6.1, suggesting mild enhancement with minimal capsule inclusion. Notably, samples C (7.3), D (8.0), and E (8.5) demonstrated progressively stronger preference, with Sample E achieving the highest level of acceptability. This result highlights the synergistic contribution of encapsulated lemon essential oil to the cream’s organoleptic appeal. Hedonic analysis confirmed that increasing concentrations of lemon essential oil-loaded pectin capsules enhanced the perceived quality of the cream, particularly in terms of mouthfeel and lemon aroma. Both ANOVA and PCA validated the sensory improvements, supporting the use of this encapsulation approach as a promising strategy to develop functional, appealing dairy products. A principal component analysis (PCA) was conducted to visualize the multidimensional sensory data and identify clustering patterns among formulations. The first two principal components, PC1 and PC2, explained 79.8% of the total variance (53.0% and 26.8%, respectively), ensuring robust representation of the sensory variability. As shown in Figure 5, distinct groupings emerged: Samples A and B were associated with lower aromatic intensity and more fluid or neutral texture profiles, whereas samples C, D, and E clustered strongly with such descriptors as *creamy*, *smooth*, and *lemon-scented*. These clusters are further supported by confidence ellipses, which visually confirm the reproducibility and consistency within each group. The PCA thus highlights the positive influence of capsule concentration on texture and olfactory expression, complementing the findings of the ANOVA.

## 4. Discussion

The design and characterization of lemon essential oil-loaded pectin capsules represent a central innovation of this study. The ultrasound-assisted ionotropic gelation method allowed the formation of uniform capsules with smooth morphology and high encapsulation efficiency (78.4%), while maintaining compound stability and controlled release properties. These features directly contributed to the improved safety, stability, and sensory quality of the cream matrix. The present study demonstrates that the post-pasteurization incorporation of lemon essential oil (LEO)-loaded pectin capsules into cream enhances multiple quality dimensions, including textural, microbiological, and sensorial attributes. Unlike conventional flavoring or preservation methods, this encapsulation strategy allows for the protection of volatile bioactives, such as limonene and citral, which are otherwise sensitive to heat and prone to degradation during dairy processing. Our approach confirms previous findings by de Souza et al. [8], who observed the thermal instability of essential oil components and the advantages of post-thermal incorporation for preserving their bioactivity in dairy matrices. In our case, incorporation at temperatures below 35 °C preserved capsule integrity and maintained the rheological profile of the cream, which benefited from the structuring role of the pectin network. As observed in related work on pectin-stabilized emulsions in probiotic yogurt [40], hydrocolloids act as texturizing agents by enhancing water-binding capacity and forming a cohesive gel matrix that prevents phase separation. In our trials, viscosity improved proportionally with capsule concentration, without negatively impacting mouthfeel or appearance.

The antimicrobial properties of the LEO-loaded capsules further support their technological relevance. Dose-dependent inhibition of *E. coli* and *S. aureus* was observed, with activity approaching that of pure essential oil at the highest loading (1%). Importantly, this antibacterial efficacy was retained over 28 days of cold storage, during which the formulated creams consistently outperformed controls in terms of microbial safety, pH stability, and structural integrity. These findings resonate with the encapsulated thyme oil system, which showed strong antibacterial activity in dairy matrices [43] while avoiding off-flavors and textural defects [44]. Our results similarly suggest that the pectin capsule acts not only as a physical barrier but also as a release modulator for bioactive volatiles, which diffuse gradually through the matrix, extending the period of microbial protection. Moreover, the sustained low counts of Enterobacteriaceae and coagulase-positive Staphylococcus in the enriched creams, even after 21 days, point to an effective hurdle technology suitable for extending shelf-life without synthetic preservatives.

From a sensory standpoint, both the ranking and hedonic tests confirmed that capsule enrichment enhanced the perceived quality of the cream, particularly in terms of creaminess and fresh lemon aroma.

While no statistically significant differences were detected in the paired comparison tests, indicating an absence of sensory defects, the hedonic scores improved steadily, with the 0.75% and 1% formulations rated highest by panelists. The PCA further revealed strong correlations between higher capsule concentrations and desirable sensory descriptors, such as “smoothness” and “aromatic freshness”. The PCA analysis clearly illustrated the clustering of samples C, D, and E along the first principal component (PC1), which explained 53.0% of the total variance. This grouping suggests that higher concentrations of lemon essential oil capsules are consistently associated with more desirable sensory traits. In contrast, samples A and B grouped along negative PC1 values, aligning with lower hedonic scores and muted flavor profiles.

This sensory enhancement is likely attributable to the controlled release behavior of encapsulated LEO, which protects volatile compounds and facilitates gradual diffusion through the cream matrix [45]. Such delayed aroma release could be responsible for the more persistent and pleasant perception of lemon notes noted in enriched samples. The statistically significant increase in hedonic scores across samples C to E reflects a concentration-dependent relationship between encapsulated LEO and perceived product quality. These findings confirm the acceptability threshold lies between 0.5% and 0.75%, a range where aroma intensity and mouthfeel are both optimized.

Pectin’s contribution to creaminess and viscosity was reinforced by the sensory panel’s feedback and by the PCA loadings, where “smoothness” and “cohesiveness” loaded heavily on PC1. These textural descriptors not only enhanced the cream’s mouthfeel but also contributed to flavor retention by reducing matrix fluidity. Our results are in line with previous studies on citrus-based encapsulation in dairy systems but uniquely demonstrate that even simple pectin-based ionotropic capsules, without synthetic stabilizers, can achieve dual functionality, i.e., preserving flavor and improving texture [11,46].

From a technological and commercial perspective, the use of lemon essential oil-loaded pectin capsules in cream production offers a scalable and clean-label innovation. The ionotropic gelation technique employed is relatively simple, cost-effective, and adaptable to existing dairy processing lines, especially given that incorporation occurs post-pasteurization and does not require specialized high-energy equipment. Unlike more complex nanoencapsulation approaches involving synthetic surfactants or high-pressure homogenization, this method is consistent with the principles of green chemistry and minimal processing, making it attractive for both small- and large-scale dairy manufacturers. Moreover, the encapsulation system simultaneously enhances product safety, sensory quality, and shelf-life, features that respond to key consumer demands in the functional and natural dairy sectors.

The strong performance of the 0.75–1% formulations suggests an optimal range for formulation that maximizes consumer acceptance without compromising product stability or cost-efficiency. Given the rising interest in plant-based preservatives and flavor systems, particularly in the Middle Eastern, North African, and European markets, this technology holds promise for extension to other dairy applications, such as yogurt, cheese spreads, or fermented beverages. Furthermore, enhanced antimicrobial protection may enable mild preservation strategies or reduced cold chain requirements, potentially lowering logistical costs and food waste, factors of growing relevance in the sustainability-driven food industry.

## 5. Conclusions

This study demonstrated that the post-pasteurization enrichment of fermented-free cream with lemon essential oil-loaded pectin capsules represents a promising clean-label strategy for enhancing both the functional and sensory qualities of dairy products. The ionotropic gelation process, applied to low methoxyl pectin and optimized through ultrasound-assisted emulsification, enabled effective encapsulation of volatile aromatic compounds while preserving their bioactivity during storage. The addition of capsules improved physicochemical parameters, particularly viscosity, total solids, and phase stability, without altering the cream’s baseline composition. Microbiological analyses confirmed that the capsules exerted a protective antimicrobial effect, notably against *E. coli* and *Staphylococcus aureus*, and maintained the safety of the product during 28 days of refrigerated storage. Sensory evaluations, especially ranking and hedonic scoring, highlighted a significant increase in consumer acceptability, while the paired comparison test confirmed the absence of disruptive changes in aroma perception.

Future studies should explore the applicability of this encapsulation system in fermented products, low-fat creams, and plant-based alternatives. Additionally, detailed characterization of essential oil release kinetics during storage and simulated digestion is needed to better understand their in vivo functionality and bioavailability.

In conclusion, the use of essential oil-loaded pectin capsules provides a dual benefit of sensory enhancement and potential microbial protection while maintaining a natural, additive-free formulation. Although the lower microbial counts observed in treated samples are consistent with the antimicrobial contribution of lemon essential oil, the present study did not include intentional inoculation with pathogenic microorganisms; therefore, this effect cannot be confirmed without further challenge testing. Nevertheless, this technology offers market-ready potential for producers seeking to differentiate products through natural flavor delivery, clean-label positioning, and improved shelf-life, responding to current consumer demand for minimally processed and functionally enriched dairy products.

## Figures and Tables

**Table 1:** Diameter of inhibition zones (mm) for pectin capsules loaded with lemon essential oil.

LEO Concentration in Capsules	*E. coli* (mm)	*S. aureus* (mm)
0% (control)	0.0 ± 0.0	0.0 ± 0.0
0.25%	7.2 ± 0.3	9.1 ± 0.4
0.5%	10.4 ± 0.5	12.8 ± 0.3
0.75%	13.5 ± 0.6	16.2 ± 0.4
1.0%	15.1 ± 0.4	18.9 ± 0.5
Pure LEO (control)	18.3 ± 0.5	22.1 ± 0.3

Values represent mean inhibition zone diameters (mm) ± standard deviation (*n* = 3). Pure lemon essential oil (LEO) was used as the positive control, while blank pectin capsules (0%) served as the negative control. Inhibition zones were measured after 24 h of incubation at 37 °C using the agar well diffusion method against *Escherichia coli* ATCC 25922 and *Staphylococcus aureus* ATCC 25923.

**Table 2:** Physicochemical parameters of cream samples at different processing stages.

Processing Stage	pH	Titratable Acidity (°D)	Apparent Viscosity (mPa·s)	Total Solids (%)
Before pasteurization	6.68 ± 0.02	16.5 ± 0.5	1230 ± 15	37.85 ± 0.12
After pasteurization	6.58 ± 0.03	17.0 ± 0.4	1315 ± 18	37.60 ± 0.10
After addition of 0.25% capsules	6.50 ± 0.02	17.8 ± 0.3	1450 ± 20	38.20 ± 0.15
After addition of 0.5% capsules	6.45 ± 0.02	18.2 ± 0.3	1625 ± 22	38.45 ± 0.13
After addition of 0.75% capsules	6.40 ± 0.02	18.5 ± 0.4	1780 ± 25	38.70 ± 0.11
After addition of 1.0% capsules	6.35 ± 0.01	19.0 ± 0.3	1920 ± 28	39.10 ± 0.14

Values are expressed as mean ± standard deviation (I = 3). Titratable acidity is expressed in Dornic degrees (°D), where 1 °D corresponds to 0.1 mL of 0.1 N NaOH. Apparent viscosity was measured at 4 °C using a rotational viscometer. Total solids were determined by gravimetric analysis after drying. Increases in capsule concentration correspond to higher viscosity and solids content, indicating improved matrix structuring.

**Table 3:** Stability monitoring of cream samples during refrigerated storage (0–28 days).

Day	Sample	pH	Viscosity * (mPa·s)	Phase Separation	Enterobacteria (CFU/g)	Coagulase+ Staph. (CFU/g)	Salmonella (25 g)	Listeria (25 g)
0	Control	6.58	1230	Absent	<10	Absent	Absent	Absent
	Formulated	6.50	1450	Absent	<10	Absent	Absent	Absent
3	Control	6.48	1225	Absent	<10	Absent	Absent	Absent
	Formulated	6.48	1445	Absent	<10	Absent	Absent	Absent
7	Control	6.44	1200	Very slight	<10	Absent	Absent	Absent
	Formulated	6.46	1400	Absent	<10	Absent	Absent	Absent
10	Control	6.25	1000	Initial separation	<10	15	Absent	Absent
	Formulated	6.40	1380	Absent	<10	Absent	Absent	Absent
14	Control	6.00	950	Visible separation	10–25 **	50	Absent	Absent
	Formulated	6.38	1340	Absent	<10	Absent	Absent	Absent
21	Control	5.80	900	Marked separation	10–25 **	80	Absent	Absent
	Formulated	6.30	1320	Slight separation	<10	30	Absent	Absent
28	Control	5.40	800	Severe separation	10–25 **	150	Absent	Absent
	Formulated	6.25	1300	Significant separation	<10	50	Absent	Absent

Data are expressed as mean values (*n* = 3). “Phase separation” refers to visible separation of the aqueous phase. Formulated samples contained 1% (*w*/*w*) lemon essential oil-loaded pectin capsules. No *Salmonella* or *Listeria monocytogenes* were detected in any sample over the 28-day refrigerated storage. * Parameters for viscosity and phase separation are included to assess physical stability and product quality, independent of microbial growth outcomes. ** Counts reported as 10–25 CFU/g indicate enumeration values above the detection limit but within the lower quantifiable range of the method used.

**Table 4:** Results of the paired comparison test (*n* = 10 panelists).

Pair	1st Preferred	2nd Preferred	χ^2^ Calculated	Critical Value (α = 0.05)	Conclusion
A vs. B	2	8	3.6	9	n.s.
A vs. C	1	9	6.4	9	n.s.
A vs. D	1	9	6.4	9	n.s.
A vs. E	1	9	6.4	9	n.s.
B vs. C	3	7	1.6	9	n.s.
B vs. D	2	8	3.6	9	n.s.
B vs. E	1	9	6.4	9	n.s.
C vs. D	4	6	0.8	9	n.s.
C vs. E	4	6	0.8	9	n.s.
D vs. E	4	6	0.8	9	n.s.

Each pairwise comparison was evaluated by 10 trained panelists. Chi-square (χ^2^) values were calculated based on observed frequencies. The critical value for significance was 9 (α = 0.05, df = 1). n.s. = no significant difference.

**Table 5:** Ranking test results for cream samples enriched with lemon essential oil-loaded pectin capsules.

Panelist	A (0%)	B (0.25%)	C (0.5%)	D (0.75%)	E (1%)
1	5	4	2	1	3
2	5	4	1	2	3
3	5	3	2	1	4
4	5	4	2	1	3
5	5	4	2	1	3
6	5	4	2	1	3
7	5	4	1	2	3
8	5	4	3	2	1
9	5	4	2	1	3
10	5	4	3	2	1
Total	50	39	20	14	27

Panelists ranked five cream samples from 1 (most preferred) to 5 (least preferred). Lower total scores indicate higher overall preference. Sample A corresponds to the control (0% capsules), while samples B–E were enriched with increasing concentrations of lemon essential oil-loaded pectin capsules. Data were analyzed using Friedman’s test (α = 0.05), revealing significant differences among samples.

## Data Availability

The original contributions presented in the study are included in the article/Supplementary Materials. Further inquiries can be directed to the corresponding author.

## Supplementary Materials

The following supporting information can be downloaded at: https://www.mdpi.com/article/10.3390/foods14162828/s1, Table S1. Hedonic test form used for sensory evaluation of treated and control cream samples; Table S2. Paired comparison test bulletin for sensory evaluation of aroma, texture, and preference; Table S3. Ranking test form for sensory preference of cream samples enriched with encapsulated lemon essential oil.
