# Supplementary material for: Cream Formulated with Lemon Essential Oil-Loaded Pectin Capsules: Effects on Microbiological Quality and Sensory Properties
Source: Foods. 2025 Aug 15;14(16):2828. doi: 10.3390/foods14162828 (PMC12385489; doi:10.3390/foods14162828)
Supplement: Supplementary file 1 [file foods-14-02828-s001.zip › foods-3753216-supplementary.pdf]

Enhancing the Sensory and Microbiological Quality of Cream Using Pectin Capsules Loaded with Lemon Essential Oil

Djerri Rofia[1], Merniz Salah[2], Himed Louiza[1*], Maria D'Elia[3,4,5] , Luca Rastrelli[3,4*]

[1] The Biotechnology and Food Quality Research Laboratory (BIOQUAL), INATAA, University Freres Mentouri Constantine 1, Algeria
[2] Institute of Industrial Hygiene and Safety, University Batna 2, Batna 05078, Algeria;
[3] Department of Pharmacy, University of Salerno, Via Giovanni Paolo II, 132, 84084Salerno, Italy
[4] National Biodiversity Future Center (NBFC), 90133 Palermo, Italy;
[5] Dipartimento di Scienze della Terra e del Mare, University of Palermo. Palermo, Italy
*Correspondence: louiza.himed@umc.edu.dz (L.H.); rastrelli@unisa.it (L.R.)

Supplementary Materials

Table S1. Hedonic Test Form Used for Sensory Evaluation of Treated and Control Cream Samples

NAME: ________________________ Panelist No.: ______

Please examine and taste each cream sample, and assign a score from 1 to 9 based on the intensity of the characteristic.

.

		**Samples**				
		**A**	**B**	**C**	**D**	**E**
**Texture**	Liquid					
	Fatty					
	Creamy					
	Smooth					
	Fluid					
	Fine					
	Supple					
	Coating					
**Color**	Yellow					
	Fresh cream					

**Odor**	Green grass					
	Dried fruit					
	Lemon					
	Rancid					
**Taste**	Acidic					
	Salty					
	Sweet					
	Bitter					
**Aroma**	Fresh cream					
	Lemon					
	Hazelnut					
	Raw milk					
	Dried fruit					

Note: If the attribute mentioned in the data sheet is not detected in the product, enter 0. The score should be assigned based on the intensity of the taste.

Table S2. Paired Comparison Test Bulletin for Sensory Evaluation of Aroma, Texture, and Preference

Date:<br><br>Name:<br><br>Taster number:<br><br>Purpose of the test:<br><br>Criteria being tested:				
Pairs for testing	Is there a difference?	Which sample has the strongest flavour?	Which sample is the creamiest?	Which sample is your favourite?

Code n°<br>/code n°<br>.......<br>…….<br>……<br>……..<br>…….<br>……..<br>……..<br>……..<br>……..<br>……..<br>……...<br>……..<br>………<br>……...<br>……..<br>……...<br>……..<br>……...<br>………<br>……..	Yes No<br>Yes No<br>Yes No<br>Yes No<br>Yes No<br>Yes No<br>Yes No<br>Yes No<br>Yes No<br>Yes No	……………………<br>…………………….<br>……………………<br>……………………..<br>………………………<br>……………………….<br>……………………….<br>………………………<br>……………………….<br>………………………..	……………………<br>…………………….<br>……………………<br>……………………..<br>………………………<br>……………………….<br>……………………….<br>………………………<br>……………………….<br>………………………..	……………………<br>…………………….<br>……………………<br>……………………..<br>………………………<br>……………………….<br>……………………….<br>………………………<br>……………………….<br>………………………..
Comments :………………………………………………………………………………………………………<br>…………………………..				

Table S3. Ranking Test Form for Sensory Preference of Cream Samples Enriched with Encapsulated Lemon Essential Oil

FICHE DE TEST DE CLASSEMENT

NAME :……………………….. N° Taster: …………..

Please rank the four samples in order of preference Date: …………..

.

Code	Ranking
……..	…………..
………	…………..
……..	……………
……..	…………..
………	…………..
